# Combined and isolated effects of workstation ergonomics and physiotherapy in improving cervicogenic headache and work ability in office workers: a single-blinded, randomized controlled study

**DOI:** 10.3389/fpubh.2024.1438591

**Published:** 2024-11-28

**Authors:** Gopal Nambi, Mshari Alghadier, Shahul Hameed Pakkir Mohamed, Arul Vellaiyan, Elturabi Elsayed Ebrahim, Dena Eltabey Sobeh, Faizan Z. Kashoo, Alaa Jameel A. Albarakati, Naif A. Alshahrani, Vijayamurugan Eswaramoorthi

**Affiliations:** ^1^Department of Health and Rehabilitation Sciences, College of Applied Medical Sciences, Prince Sattam bin Abdulaziz University, Al-Kharj, Saudi Arabia; ^2^Department of Health Rehabilitation Sciences, Faculty of Applied Medical Sciences, University of Tabuk, Tabuk, Saudi Arabia; ^3^Saveetha College of Physiotherapy, Saveetha Institute of Medical and Technical Sciences (Deemed to the University), Chennai, TamilNadu, India; ^4^College of Nursing, Prince Sattam bin Abdulaziz University, Al Majmaah, Saudi Arabia; ^5^Department of Physical Therapy and Health Rehabilitation, College of Applied Medical Sciences, Majmaah University, Al Majmaah, Saudi Arabia; ^6^Department of Surgery, College of Medicine, Umm Al-Qura University, Al-Qunfudah Branch, Makkah, Saudi Arabia; ^7^Orthopedic Surgery Department, King Fahad Medical City, Ministry of Health, Riyadh, Saudi Arabia; ^8^Department of Physiotherapy, Faculty of Pharmacy and Health Sciences, Royal College of Medicine Perak, Universiti Kuala Lumpur, Ipoh, Malaysia

**Keywords:** cervicogenic headache, ergonomics, workstation, physiotherapy, office workers

## Abstract

**Objective:**

The objective of the study is to compare and investigate the combined and individual effects of workstation ergonomics, physiotherapy and patient education in improving CgH headaches and work ability in office workers.

**Methods:**

96 eligible CgH participants were divided into the ergonomics modifications group (EMG; *n* = 24), physiotherapy group (PTG; *n* = 24), and ergonomics modifications combined with physiotherapy group (EPG; *n* = 24) and education control group (CNG; *n* = 24), the participants received the respective treatment for 4 weeks. Primary (CgH frequency) and secondary (CgH pain intensity, CgH disability, flexion rotation test (right and left), neck disability index and work ability) scores were measured. The effects of treatment at various intervals were analyzed with a 4 × 4 linear mixed model analysis (LMM) between treatment groups and time intervals.

**Results:**

Four weeks following training EPG group showed more significant changes in primary outcome CgH frequency; 4.6 CI 95% 3.63 to 5.56 when compare to control group. The same gradual improvement was noticed at 8 weeks 8.2 CI 95% 7.53 to 8.86 and at 6 months follow up 11.9 CI 95% 11.25 to 12.54 when compare to other groups (*p* = 0.001) which is statistically 52.97% improvement. Similar improvements can be seen in the secondary outcome measures such as CgH pain intensity, CgH disability, flexion rotation test (right and left), neck disability index and work ability in EPG group than the EMG, PTG, and CNG groups (*p* = 0.001) at 4 weeks, 8 weeks and at 6 months’ follow-up.

**Conclusion:**

This study observed that the workstation ergonomics and physiotherapy group experienced significantly more improvements in cervicogenic headache patients.

**Clinical trial registration:**

Identifier NCT05827185

## 1 Introduction

According to the Global Burden of Disease (GBD) survey, headache disorders affect approximately 66% of the general population aged between 20 and 85 years. In addition, 44.4% of men and 57.8% of women report experiencing headaches at least once in their lifetime (1). Cervicogenic headache (CgH) is a distinct form of headache and accounts for 15–20% of all headaches, with a prevalence rate ranging from 0.4 to 20% (2). The prevalence of CgH is 0.21% in females and 0.13% in males, with office workers being more affected than other occupations (3). The International Classification of Headache Disorders, 3rd edition (ICHD-3), is a globally recognized system used to classify and diagnose headaches. It categorizes headaches into three main types: primary headaches, secondary headaches, and other headache disorders. The ICHD-3 diagnostic criteria for cervicogenic headache identify it as a headache originating from a disorder of the cervical spine or neck tissues. Diagnosis requires evidence of a cervical lesion that explains the headache, along with at least two of the following: onset in relation to the cervical disorder, improvement after treatment of the cervical disorder, or provocation by neck movements or pressure. Additionally, the headache must not be better explained by any other headache disorder (4). Poor workstations and bad posture during work are generally the main causes of cervicogenic headache. The cause of CgH is located in the neck region, and the pain worsens with asymmetrical movements of the head and neck (5). The most accepted mechanism of CgH involves the interaction between the trigeminal nerve and the C1–C3 nerves in the trigeminal-cervical nucleus (6). CgH usually arises from musculoskeletal structures such as the cervical vertebrae, intervertebral discs, or paravertebral muscles. The clinical features of CgH include unilateral headache, limited range of motion (ROM) in the neck, and referred pain to the head or face (7).

Cervicogenic headache (CgH) is generally diagnosed based on a detailed history and clinical assessment (8). Clinical examinations typically reveal pain in the cervical region, including neck pain (NP), decreased neck movements, upper-quarter muscle tightness, and loss of muscle function (9). The flexion-rotation test (FRT) is a valid and reliable method for assessing neck movements and is recognized as a diagnostic tool for CgH (10).

The management of CgH involves both pharmacological and non-pharmacological methods, with pharmacological approaches often associated with many side effects (11). There are several non-pharmacological treatment options available, such as ergonomic modifications and guidance, physiotherapy, acupuncture, massage, dry needling, and patient education (12, 13).

The Guide to Health and Safety in the Office handbook by the Commonwealth of Australia (14) suggests ergonomic guidance and interventions for preventing and treating musculoskeletal disorder (MSD) injuries in office workers. To date, there is no scientific evidence from randomized control trials specifically examining the application of these interventions for preventing and treating cervicogenic headache in office workers. Also, studies exploring the effects of ergonomic interventions on neck pain have produced mixed results. Tsang et al. (15) and Lee et al. (16) provide strong evidence supporting the effects of integrated ergonomic interventions and motor control exercises on muscle activity and kinematics in people with work-related neck and shoulder pain. Van Eerd et al. found moderate evidence for the effectiveness of job stress management training and office workstation adjustments in reducing upper extremity MSD and its symptoms (17). Hoe et al. conducted a systematic review and meta-analysis on CgH and found inconsistent evidence for the use of ergonomic modifications to reduce the incidence of neck or shoulder pain (18).

It is estimated that 34% of US citizens receive some form of physiotherapy for cervicogenic headache (CgH) each year (19). In physiotherapy, physical modalities such as infrared radiation (IRR), shortwave diathermy (SWD), transcutaneous electrical nerve stimulation (TENS), interferential therapy (IFT), and hydro collator pack application, along with muscle strengthening exercises, joint mobilization and manipulation techniques, and postural correction exercises, are commonly used to treat CgH patients (20). In addition, during patient education, therapists spend time with patients to improve their overall health. The educator considers each patient’s abilities and needs, and interacts with them accordingly. This approach enhances patients’ self-efficacy, self-health management, health knowledge, health awareness, and overall well-being (21).

Till date, no studies have compared and investigated the combined and individual effects of workstation ergonomics, physiotherapy, and patient education on improving cervicogenic headache (CgH) and work ability in office workers. Moreover, current studies do not address the shortcomings and gaps in the existing literature on managing CgH in office workers, such as the lack of comparisons between intervention procedures, inadequate trial designs, poor study methods, and small sample sizes. Therefore, our study aims to compare and investigate the combined and individual effects of workstation ergonomics, physiotherapy, and patient education in improving primary (CgH frequency) and secondary outcomes (CgH pain intensity, CgH disability, flexion rotation, and work ability) in office workers with cervicogenic headache. This randomized clinical trial hypothesizes that there is a difference in primary (CgH frequency) and secondary (CgH pain intensity, CgH disability, flexion rotation, and work ability) outcome measures between workstation ergonomics, physiotherapy, and patient education in office workers with cervicogenic headache.

## 2 Methods

### 2.1 Study design

The trial was a prospective, single-blinded, parallel-group, randomized controlled trial. The required participants were screened by an orthopedic surgeon at the University Hospital between May 2020 and February 2023, following the cervicogenic headache 11.2.1 from the ICHD-3 (International Classification of Headache Disorders-3) classification (4). Ninety-six (*N* = 96) participants who fulfilled the eligibility criteria were randomly allocated into four groups equally: the ergonomics modifications group (EMG; *n* = 24), the physiotherapy group (PTG; *n* = 24), the ergonomics modifications combined with the physiotherapy group (EPG; *n* = 24), and, education control group (CNG; *n* = 24) through a computer-generated block random table (blocks of four) and the allocation of the participants to each group was concealed using sealed envelopes. The computer did not generate the group until it was time to randomize each participant, ensuring that the allocation was concealed. No significant changes were made while the study was being carried out because it was designed as a follow-up to a pilot study.

The research was conducted at Out-patient physiotherapy clinic, Department of Physical therapy and Health Rehabilitation, Prince Sattam bin Abdulaziz University, Al Kharj, Saudi Arabia, and the Department Ethical Committee (DEC) granted ethical approval under the reference number RHPT/019/082. The DEC accepted the study protocol as well as the informed consent forms. The study followed the instructions outlined in the Declaration of Helsinki (1964) and was retrospectively registered in the clinical trial registry NCT05827185 on April 23, 2023.

### 2.2 Participants

**Figure 1:**
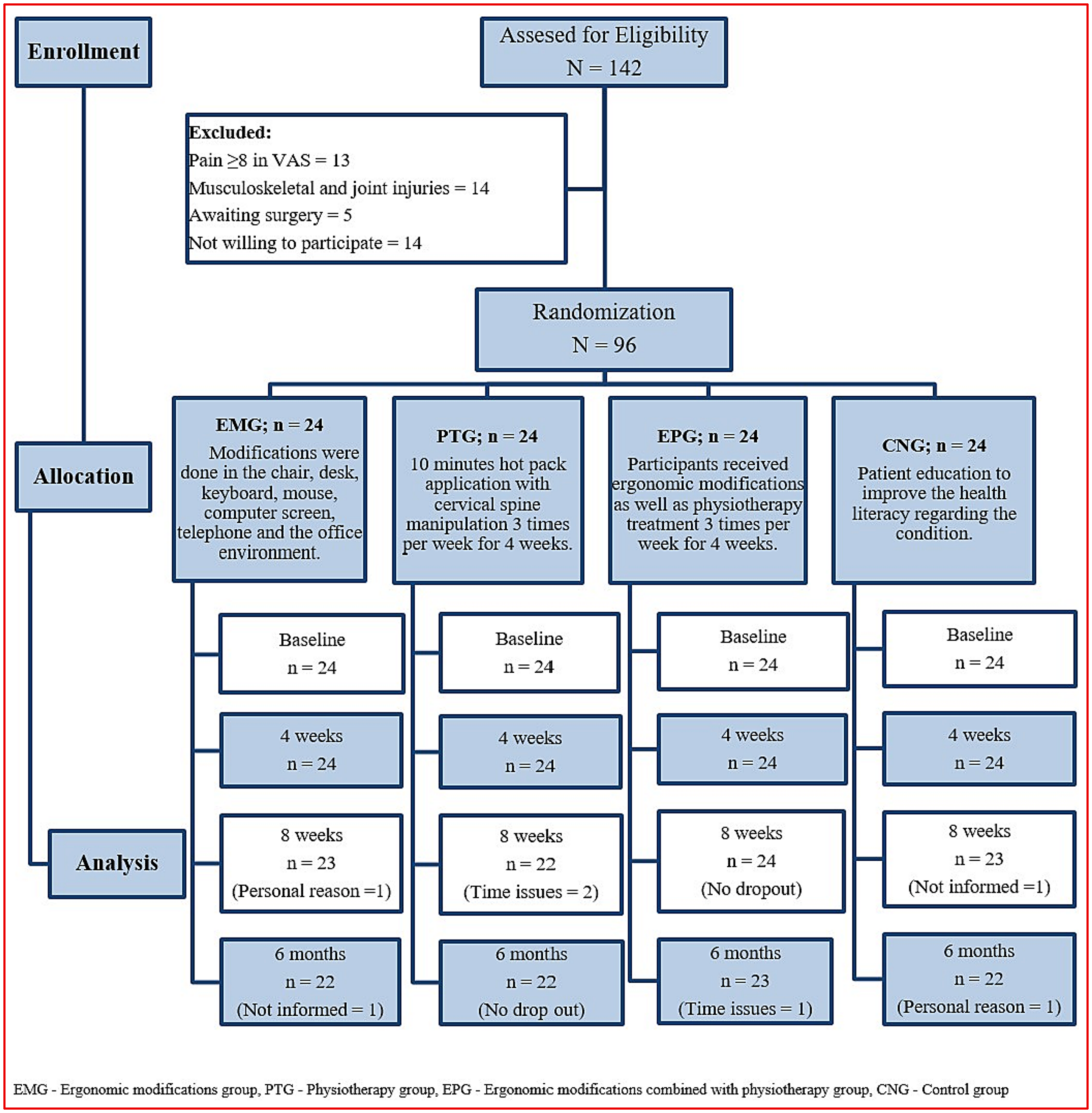
Flow chart showing the study details.

Patients aged between 18 and 58 years, working on the computer 32 h per week and suffering from CgH (>3 months) were screened to be included in the study. They were diagnosed based on the diagnostic criteria developed by the ICHD-3 by an orthopaedic surgeon with twenty years of clinical experience in diagnosing and treating the CgH condition. The problem of cervicogenic headache falls under the International Classification of Disease-10 (ICD-10) code of G44. 841 (8). Patients with pain intensity of 3 or more on a numerical pain rating scale (NPRS), CgH resulting from pain in the neck followed by headache, limited neck movements, neck muscle spasm, cervical spine disorders and those consenting to participate in the study were included in the study. Other primary headaches such as migraine and tension-type headaches (TTH), whiplash injuries, participants who show signs of the five ‘D’s’ (dizziness, drop attacks, dysarthria, dysphagia, diplopia) or who had signs of the three ‘N’s (nystagmus, nausea, other neurological symptoms (cord compression or nerve root involvement), contraindications to physiotherapy (congenital anomalies, tumors, degenerative and inflammatory arthritis, osteoporosis, dislocation, fractures, and steroid intake), underwent previous head and neck surgeries or other complementary therapies in the last 3 months were excluded. The flow of the study program was documented following the CONSORT guidelines and displayed in Figure 1).

The list of participants was compiled from five government sectors and 13 private companies and requests were sent to the organizations and participants via personal e-mail from the research team. Participants and their organizations were not reimbursed for their participation in the research. The research was carried out at the Department of Physical Therapy and Health Rehabilitation, Prince Sattam bin Abdulaziz University, Al Kharj, Saudi Arabia, using the recommended study protocols.

### 2.3 Interventions

Four certified physiotherapists with 10–15 years of experience in providing ergonomic advice and providing physiotherapy for CgH patients were included in the research group. All the participants in the four groups expressed their willingness to participate in the research after getting detailed information about the study protocol. First of all, the participant’s neck muscles and cervical joints were assessed for any musculoskeletal dysfunction and the complaints were noted. After that, the participants in each group received their appropriate interventions as per the directions of the study protocol. Standardized treatment techniques were used for each group of participants to reduce intervention bias. The procedures for intervention and follow-up measurements were recorded in standardized forms. During the study period, the participants were asked to refrain from taking any other type of intervention, they received the concerned interventions 3 times a week for 4 weeks.

#### 2.3.1 Ergonomics modifications

**Figure 2:**
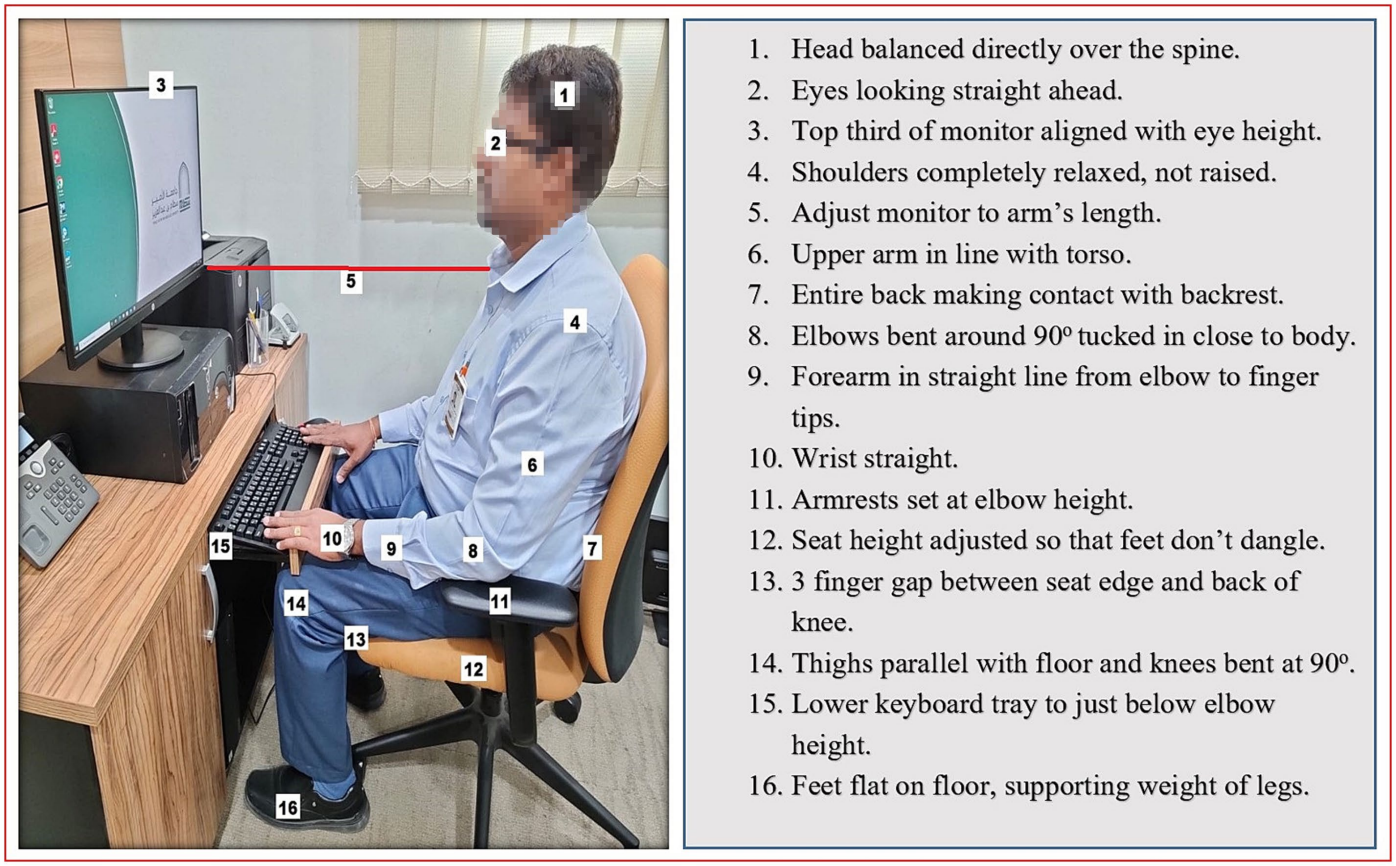
Figure showing the workstation ergonomics.

All the participants in the EMG and EPG underwent the workstation assessment in an individualized manner. The assessment was done during working hours with special permission from the higher authorities. A blinded therapist used an observation-based ergonomics assessment checklist for office workers to check the status of the office environment, which usually took 40–45 min to complete. It consists of 5 domains (7 items related to office chairs, 9 items related to office desks, 8 items related to keyboard and mouse, 5 items related to the computer screen, 3 items related to telephone and 5 items related to the office environment) and it has good reliability and validity (22). After assessment, the required modifications were done in the chair, desk, keyboard, mouse, computer screen, telephone and the office environment (Figure 2). Ergonomics education and instructions were also given on an individual basis as per the report of the assessment.

#### 2.3.2 Physiotherapy

All the participants in the PTG and EPG received the standard physiotherapy as per the approved study protocol. Four experienced physiotherapists having experience in treating CgH provided physiotherapy after the evaluation of each participant. First, a hydro collator pack was applied over the neck region for 10 min to relax the muscles of the neck region. Then the therapist identified the sites of abnormal changes in each vertebra and cervical manipulation was given. To perform the C1-C2 cervical spine manipulation (CSM), the participant was instructed to lie in a face-up position with upper and lower extremities kept aside relaxed. The head was kept in a neutral position and the treating therapist stands at the patient’s head side and holds the chin of the patient with the right hand. The therapist’s left hand holds the posterior aspect of the head and performs two to three free rotatory movements. Afterwards, the therapist performs the (High velocity low amplitude thrust – HVLAT) technique either the right or left direction based on the symptoms provided by the patient. The manipulation was first done on the pain-free side and then on the painful side and the rotation range is limited by the target vertebra. If any participant reported any new red flag signs or showed no signs for manipulation, such as no pain or musculoskeletal dysfunction, then the procedure was not performed. The cervical manipulation was performed 3 times a week for 4 weeks (23).

#### 2.3.3 Patient education

Participants in the control (CNG) group received patient education from an experienced physiotherapist, each session lasted for 30 min for 4 weeks in small groups consisting of 8–10 participants. According to each patient’s abilities, the therapist educated them to improve their health literacy regarding the condition. The therapist educated them about the benefits of self-care activities, maintaining good posture, staying active and doing active movements and stretching muscles for preventing health-related musculoskeletal injuries at the workstation. Also, lifestyle modifications were taught to the patients to prevent further deterioration of the condition.

Participants of all four groups were asked to perform neck isometric exercises three times a day, for every day. In which, the patient was asked to keep his hand over his forehead and resist the forward movement of his neck for 10 s and the same movement was repeated 15 times. Similarly, the patient was asked to keep the hand on the posterior and lateral sides of the head and resist the backward and sideways movements of the neck. Also, static active stretching exercises for the upper trapezius, levator scapulae, scalene, and sternocleidomastoid muscles were taught to the patients, which was maintained for the 30s with 3 repetitions. The patients were instructed to keep doing this set of exercises after 4 weeks of various intervention protocols and they were asked to maintain an exercise log book to check the treatment compliance.

### 2.4 Outcomes

All the outcome measures were recorded by a blinded physiotherapist, and the scores were entered in a data sheet. The scores were measured at the beginning of the study, after 4-weeks, 8-weeks, and at 6-months.

#### 2.4.1 Primary outcome

##### 2.4.1.1 CgH frequency

It is a self-administered outcome variable where the patient enters his CgH pain experience in a medical log book every evening to find the number of painful days in 4-weeks (24).

#### 2.4.2 Secondary outcome

##### 2.4.2.1 CgH pain intensity

The pain intensity of CgH was assessed using an 11-point numerical pain rating scale (NPRS). Patients rated their typical level of pain status during the previous week on a 10 cm horizontal line, with one end 0 representing “no pain” and the other end 10 representing “worst pain imaginable” (25).

##### 2.4.2.2 CgH disability

The Headache Impact Test (HIT) questionnaire is a valid and reliable instrument to assess the level of disability in CgH patients. It consists of six items: pain, social functioning, role functioning, vitality, cognitive functioning, and psychological distress. The score categories are “no or mild disability” (49 or less), “moderate disability” (50–55), “severe disability” (56–59) and” complete disability” (60–78) (26).

##### 2.4.2.3 Cervical flexion–rotation test (FRT)

The cervical flexion–rotation test is done with the patient in a supine position. The therapist passively maintains the patient’s neck into full flexion to relax the structures of the middle and lower cervical spine, and then the patient’s head is passively rotated in each direction while the flexed position is maintained and the range of motion is measured (10).

##### 2.4.2.4 Work ability

It was measured by Work Ability Index (WAI), which consists of 7 items such as current ability, work ability about physical and mental demands of the job, reported diagnosed diseases, estimated impairment due to health status, sick leave over the last 12 months, self-prognosis of work ability in the 2 years to come and mental resources of the individual. It ranges from 7 to 49 points and 4 categories such as; “poor” (7–27), “moderate” (28–36), “good” (37–43) and “excellent” (44–49) (27).

### 2.5 Sample size

For calculating the number of subjects to be included in the study, the primary outcome measure CgH frequency in days was taken into consideration based on a previous pilot study which found the effect of physiotherapy in the treatment of CgH, with 10 subjects in each group. Using the G-Power software (version 3.1.9.2; Franz Faul, University of Kiel, Germany), assuming a two-sided *α* = 0.05, and power (1−*β* = 0.80), to detect an effect size of 1.2 CgH days and mean difference of 4 CgH days (between groups) and a standard deviation of 0.5, approximately 22 samples were required. Assuming a 10% dropout, we enrolled 24 subjects in each group.

### 2.6 Blinding

Because of the experimental nature of the study methodology, it was not possible to blind the treating therapist as well as the participants included in the study. The therapists who assessed the outcome variables at baseline, 4-weeks, 8-weeks, and 6-months were blinded. Therefore, the therapist providing the treatment and the therapist measuring the data were different individuals. In addition, the outcome-measuring therapist continued to be masked to the participant’s groups at all-time intervals. Also, participants were asked not to discuss their treatment details with their peers or the outcome-measuring therapist.

### 2.7 Statistical methods

The general count data and the normality of study participants’ demographic characteristics were analyzed through the Kolmogorov–Smirnov test. The outcome data were presented in the form of a mean and standard deviation with a 95% confidence interval. The effects of treatment at different time intervals were analyzed using a 3 × 4 linear mixed model analysis (LMM), with treatment groups (EMG, PTG, EPG and CNG) and time intervals (baseline, 4 weeks, 8 weeks, and at 6 months) and a statistical significance level of *α* = 0.05. All the statistical tests were done using GraphPad-Prism (version 9.1), Boston, MA, United States.

## 3 Results

### 3.1 Participants

Out of the 142 participants screened, 13 participants had greater than 8 scores in VAS score, 14 participants had some sort of orthopaedic and joint injuries, five participants had undergone joint surgery and 14 refused to be involved in the research and they were excluded. Therefore, ninety-six (N = 96) participants were chosen based on the eligibility criteria and allocated to one of the four groups. Two participants in the EMG (personal reason = 1 & not informed = 1), two participants in the PTG (Time issues = 2), one participant in the EPG (Time issues =1), and two participants in the CNG (not informed = 1 & personal reason = 1), did not complete the 6-month follow-up (Figure 1). The study analysis assumed the intention to treat principle method to analyze the statistical data. Overall, the compliance with follow-up data collection at 6 months was 93%, adherence to study protocols (e.g., number of visits) was 100%, and none of the participants in the four groups received any additional care that was not included in the four study interventions. At baseline, the demographic characters such as age (years), height (cm), weight (kg), and BMI (kg/m^2^) scores did not report statistically significant variation between the groups (*p* > 0.05). In all four groups, females (50–58%) are affected more than males. At baseline, the clinical variables also did not show any significant difference between groups (*p* > 0.05). The clinical presentation of headache is more unilateral (75–83%) than bilateral and the majority of CGH cases have associated neck pain (75–83%) which is shown in Table 1.

**Table 1:** Demographic details of EMG, PTG, EPG and CNG groups.

Variable		EMG	PTG	EPG	CNG	*p*-value
Age (year)	–	41.1 ± 3.9	40.8 ± 4.0	39.9 ± 3.8	40.5 ± 4.2	0.754*
Gender	Male	12 (50%)	10 (42%)	12 (40%)	11 (46%)	–
	Female	12 (50%)	14 (58%)	12 (50%)	13 (54%)	–
Height (cm)	–	165.2 ± 3.6	164.2 ± 3.8	164.8 ± 3.5	165.1 ± 3.9	0.786*
Weight (m)	–	73.88 ± 5.3	72.11 ± 4.9	74.21 ± 4.3	74.65 ± 4.1	0.259*
BMI (Kg/m^2^)	–	24.2 ± 2.18	23.9 ± 1.89	24.4 ± 2.11	23.9 ± 2.34	0.814*
CgH duration (year)	–	6.5 ± 3.2	6.6 ± 2.9	6.4 ± 3.1	5.9 ± 3.1	0.864*
CgH frequency (per day)	–	0.73 ± 0.12	0.74 ± 0.15	0.71 ± 0.11	0.75 ± 0.13	0.735*
Headache	Unilateral	18 (75%)	19 (79%)	18 (75%)	20 (83%)	–
	Bilateral	6 (25%)	5 (21%)	6 (25%)	4 (17%)	–
Neck pain	Yes	19 (79%)	18 (75%)	19 (79%)	20 (83%)	–
	No	5 (21%)	6 (25%)	5(21%)	4(17%)	–

*Non significant. EMG, Ergonomic modifications group; PTG, Physiotherapy group; EPG, Ergonomic modifications combined with physiotherapy group; CNG, Control group.

### 3.2 Primary outcome

**Figure 3:**
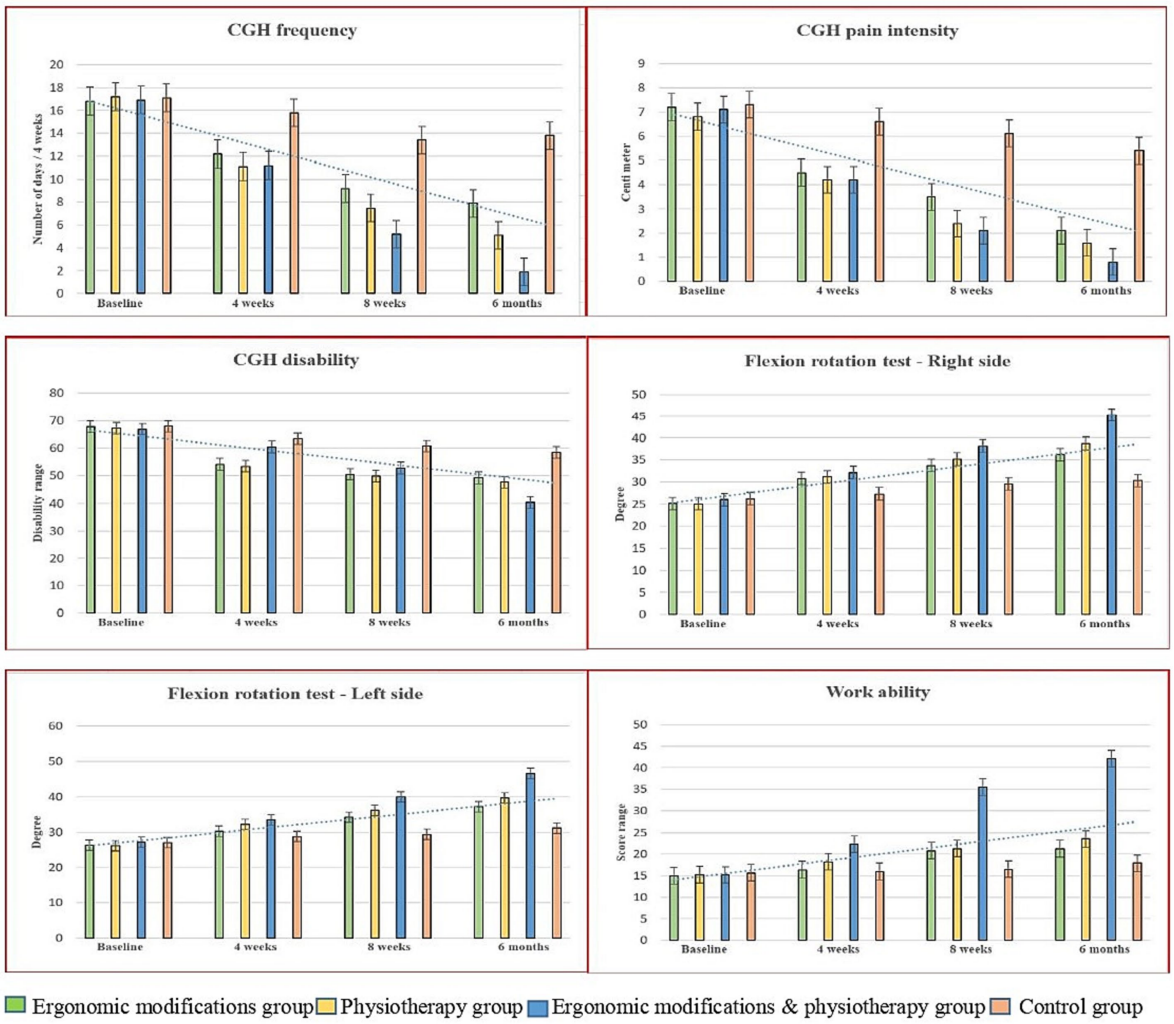
Pre and post outcome measures of EMG, PTG, EPG and CNG groups.

The baseline score on the primary outcome, CgH frequency, among the EMG, PTG, EPG, and CNG groups showed no statistical variation (*p* ≥ 0.05), indicating a homogeneous presence of study participants. The mean and standard deviation (SD) of the CgH frequency score between the four groups at four time periods appeared in Table 2. The 4 × 4 linear mixed model (LMM) analysis reported a statistically significant change in CgH frequency (*p* = 0.022, ηp^2^ = 0.152). After 4 weeks of different interventions, there was a significant change in *CgH pain frequency* between the groups: EMG vs. Control (3.6; CI 95% 2.63 to 4.56), PTG vs. Control (4.7; CI 95% 3.73 to 5.66), and EPG vs. Control (4.6; CI 95% 3.63 to 5.56) (*p* = 0.001). A similar improvement was observed at the 8-week and 6-month follow-up measurements. The posthoc Bonferroni analysis and the standard mean difference showed a higher percentage of improvement in CgH pain frequency in the EPG vs. Control group (11.9; CI 95% 11.25 to 12.54) compared to the EMG vs. Control and PTG vs. Control groups (Figure 3) at the 6-month follow-up. The complete interpretation and the effect size (d = 8.72) showed a greater effect in the EMG group, with a statistically significant 52.97% improvement in CgH frequency compared to the EMG, PTG, and Control groups. Moreover, the intra-group analysis through repeated measures ANOVA showed significant changes in all groups (*p* = 0.001).

**Table 2:** Pre and post mean and SD outcome measure scores of EMG, PTG, EPG, and CNG groups.

Variable	Duration	EMG	PTG	EPG	CNG	Group × Time *p*-value
CgH frequency (no of days per 4 weeks)	Base line	16.8 ± 1.8	17.2 ± 1.9	16.9 ± 1.7	17.1 ± 1.7	0.022 ηp^2^ = 0.152
	4 weeks	12.2 ± 1.2	11.1 ± 1.1	11.2 ± 1.4	15.8 ± 1.4	
	8 weeks	9.2 ± 0.9	7.5 ± 0.5	5.2 ± 0.9	13.4 ± 1.1	
	6 months	7.9 ± 0.7	5.1 ± 0.7	1.9 ± 0.5	13.8 ± 1.3	
	*p*-value	0.001	0.001	0.001	0.001	
CgH pain intensity (0–10)	Base line	7.2 ± 0.8	6.8 ± 0.7	7.1 ± 0.7	7.3 ± 0.6	0.012 ηp^2^ = 0.109
	4 weeks	4.5 ± 0.4	4.2 ± 0.4	4.2 ± 0.5	6.6 ± 0.6	
	8 weeks	3.5 ± 0.3	2.4 ± 0.2	2.1 ± 0.2	6.1 ± 0.6	
	6 months	2.1 ± 0.2	1.6 ± 0.1	0.8 ± 0.09	5.4 ± 0.5	
	*p*-value	0.001	0.001	0.001	0.001	
CgH Disability	Base line	67.88 ± 6.5	67.21 ± 6.8	66.91 ± 6.9	67.94 ± 6.8	0.003 ηp^2^ = 0.102
	4 weeks	54.12 ± 5.5	53.38 ± 5.6	60.38 ± 6.0	63.38 ± 6.3	
	8 weeks	50.41 ± 5.0	49.73 ± 5.2	52.67 ± 5.2	60.67 ± 6.1	
	6 months	49.19 ± 4.9	47.54 ± 4.5	40.37 ± 4.1	58.37 ± 5.8	
	*p*-value	0.001	0.001	0.001	0.001	
Flexion rotation test (Right side)	Base line	25.18 ± 7.4	25.12 ± 7.3	26.01 ± 7.2	26.22 ± 7.4	0.011 ηp^2^ = 0.141
	4 weeks	30.76 ± 6.2	31.19 ± 6.1	32.12 ± 6.5	27.32 ± 6.5	
	8 weeks	33.73 ± 5.6	35.15 ± 5.4	38.12 ± 6.1	29.52 ± 6.1	
	6 months	36.21 ± 5.2	38.71 ± 5.4	45.31 ± 4.8	30.31 ± 5.8	
	*p*-value	0.001	0.001	0.001	0.110**	
Flexion rotation test (Left side)	Base line	26.21 ± 7.3	26.12 ± 7.2	27.11 ± 7.1	27.01 ± 7.0	0.004 ηp^2^ = 0.146
	4 weeks	30.22 ± 6.1	32.14 ± 6.3	33.43 ± 6.4	28.72 ± 6.4	
	8 weeks	34.21 ± 5.6	36.11 ± 5.8	39.98 ± 5.1	29.32 ± 6.0	
	6 months	37.11 ± 5.3	39.71 ± 5.5	46.65 ± 4.6	31.19 ± 5.6	
	*p*-value	0.001	0.001	0.001	0.149**	
Work ability	Base line	15.01 ± 1.4	15.25 ± 1.5	15.21 ± 1.4	15.66 ± 1.4	0.001 ηp^2^ = 0.155
	4 weeks	16.36 ± 1.5	18.24 ± 1.8	22.32 ± 2.2	15.99 ± 1.4	
	8 weeks	20.83 ± 1.8	21.32 ± 2.2	35.53 ± 3.9	16.53 ± 1.5	
	6 months	21.28 ± 2.1	23.56 ± 2.4	42.12 ± 4.3	17.92 ± 1.7	
	*p*-value	0.001	0.001	0.001	0.001	

**Not significant. EMG, Ergonomic modifications group; PTG, Physiotherapy group; EPG, Ergonomic modifications combined with physiotherapy group; CNG, Control group.

### 3.3 Secondary outcomes

The baseline scores on the secondary outcome variables between the EMG, PTG, EPG, and CNG groups showed no statistical variation (*p* ≥ 0.05), indicating a homogeneous presence of study participants. The mean and standard deviation (SD) of the secondary outcomes between the four groups at four time periods appeared in Table 2. The 4 × 4 linear mixed model showed statistically significant variations in CgH pain intensity (*p* = 0.012, ηp^2^ = 0.109), CgH disability (*p* = 0.003, ηp^2^ = 0.102), flexion rotation test (right and left) (*p* = 0.011, ηp^2^ = 0.121, 0.146), and work ability (*p* = 0.001, ηp^2^ = 0.155).

After 4 weeks of intervention and at the 6-month follow-up, there were statistically significant variations in *CgH pain intensity* [EMG vs. Control (3.3; CI 95% 3.09 to 3.50), PTG vs. Control (3.8; CI 95% 3.59 to 4.00), EPG vs. Control (4.6; CI 95% 4.39 to 4.80)], *CgH disability* [EMG vs. Control (9.18; CI 95% 5.50 to 12.85), PTG vs. Control (10.83; CI 95% 7.15 to 14.5), EPG vs. Control (18.0; CI 95% 14.32 to 21.67)], *flexion rotation – right* [EMG vs. Control (−5.90; CI 95% −9.91 to −1.88), PTG vs. Control (−8.4; CI 95% −12.41 to −4.38), EPG vs. Control (−15.0; CI 95% −19.0 to −10.9)], *flexion rotation – left* [EMG vs. Control (−5.92; CI 95% −9.89 to −1.94), PTG vs. Control (−8.52; CI 95% −12.59 to −4.54), EPG vs. Control (−15.4; CI 95% −19.43 to −11.48)], and *work ability* [EMG vs. Control (−3.36; CI 95% −5.48 to −1.23), PTG vs. Control (−5.64; CI 95% −7.76 to −3.51), EPG vs. Control (−24.2; CI 95% −26.3 to −22.0)]. The *post hoc* Bonferroni analysis and the standard mean difference showed a higher percentage of improvement in all secondary outcome measures in the EPG group compared to the EMG and PTG groups (Figure 3). Moreover, intra-group analysis through repeated measures ANOVA for CgH pain frequency, CgH disability and work ability showed significant changes in all groups (*p* = 0.001), but not in the flexion rotation test for the right and left sides (*p* > 0.05) for the control group (see Table 3).

**Table 3:** Pre and post mean difference and confidence interval (upper limit and lower limit) scores treatment and control groups.

Variable / time		Baseline	4 weeks	8 weeks	6 months
		Mean difference (95% CI)			
CgH frequency	EMG vs. PTG	0.4 (−0.94 to 1.74)	−1.1 (−2.06 to −0.13)	−1.7 (−2.36 to −1.03)	−2.8 (−3.44 to −2.15)
	*p*-value	0.863	0.019	0.001	0.001
	EMG vs. EPG	0.1 (−1.24 to 1.44)	−1.0 (−1.96 to −0.03)	−4.0 (−4.66 to −3.33)	−6.0 (−6.64 to −5.35)
	*p*-value	0.997	0.040	0.001	0.023
	EMG vs. CNG	0.3 (−1.04 to 1.64)	3.6 (2.63 to 4.56)	4.2 (3.53 to 4.86)	5.9 (5.25 to 6.54)
	*p*-value	0.936	0.001	0.001	0.012
	PTG vs. EPG	−0.3 (−1.64 to 1.04)	0.1 (−0.86 to 1.06)	−2.3 (−2.96 to −1.63)	−3.2 (−3.84 to −2.55)
	*p*-value	0.936	0.993	0.001	0.001
	PTG vs. CNG	−0.1 (−1.44 to 1.24)	4.7 (3.73 to 5.66)	5.9 (5.23 to 6.56)	8.7 (8.05 to 9.34)
	*p*-value	0.997	0.001	0.003	0.935
	EPG vs. CNG	0.2 (−1.14 to 1.54)	4.6 (3.63 to 5.56)	8.2 (7.53 to 8.86)	11.9 (11.25 to 12.54)
	*p*-value	0.979	0.001	0.904	0.937
CgH pain intensity	EMG vs. PTG	−0.4 (−0.93 to 0.13)	−0.3 (−0.66 to 0.06)	−1.1 (−1.37 to −0.82)	−0.5 (−0.70 to −0.29)
	*p*-value	0.207	0.143	0.001	0.001
	EMG vs. EPG	−0.1 (−0.63 to 0.43)	−0.3 (−0.66 to 0.06)	−1.4 (−1.67 to −1.12)	−1.3 (−1.50 to −1.09)
	*p*-value	0.960	0.143	0.001	0.001
	EMG vs. CNG	0.1 (−0.43 to 0.63)	2.1 (1.73 to 2.46)	2.6 (2.32 to 2.87)	3.3 (3.09 to 3.50)
	*p*-value	0.960	0.001	0.035	0.937
	PTG vs. EPG	0.3 (−0.23 to 0.83)	0.0 (−0.36 to 0.36)	−0.3 (−0.57 to −0.02)	−0.8 (−1.00 to −0.59)
	*p*-value	0.455	0.001	0.026	0.001
	PTG vs. CNG	0.5 (−0.03 to 1.03)	2.4 (2.03 to 2.76)	3.7 (3.42 to 3.97)	3.8 (3.59 to 4.00)
	*p*-value	0.072	0.001	0.934	0.937
	EPG vs. CNG	0.2 (−0.33 to 0.73)	2.4 (2.03 to 2.76)	4.0 (3.72 to 4.27)	4.6 (4.39 to 4.80)
	*p*-value	0.758	0.003	0.937	0.937
CgH disability	EMG vs. PTG	−0.6 (−5.76 to 4.42)	−0.7 (−5.16 to 3.68)	−0.6 (−4.75 to 3.39)	−1.65 (−5.32 to 2.02)
	*p*-value	0.985	0.971	0.971	0.644
	EMG vs. EPG	−0.9 (−6.0 to 4.12)	6.2 (1.83 to 10.68)	2.2 (−1.81 to 6.33)	−8.82 (−12.49 to −5.14)
	*p*-value	0.959	0.002	0.470	0.001
	EMG vs. CNG	0.06 (−5.03 to 5.15)	9.26 (4.83 to 13.68)	10.26 (6.18 to 14.33)	9.18 (5.50 to 12.85)
	*p*-value	1.152	0.001	0.001	0.001
	PTG vs. EPG	−0.3 (−5.39 to 4.79)	7.0 (2.57 to 11.42)	2.94 (−1.13 to 7.01)	−7.17 (−10.84 to −3.49)
	*p*-value	0.998	0.001	0.774	0.001
	PTG vs. CNG	0.73 (−4.36 to 5.82)	10.0 (5.57 to 14.42)	10.94 (6.86 to 15.01)	10.83 (7.15 to 14.50)
	*p*-value	0.977	0.001	0.001	0.001
	EPG vs. CNG	1.03 (−4.06 to 6.12)	3.0 (−1.42 to 7.42)	8.0 (3.92 to 12.07)	18.0 (14.32 to 21.67)
	*p*-value	0.952	0.292	0.001	0.001
Flexion rotation test (right side)	EMG vs. PTG	−0.06 (−5.59 to 5.47)	0.43 (−4.34 to 5.20)	1.42 (−2.96 to 5.80)	2.5 (−1.51 to 6.51)
	*p*-value	1.118	0.995	0.831	0.366
	EMG vs. EPG	0.83 (−4.70 to 6.36)	1.36 (−3.41 to 6.13)	4.39 (0.00 to 8.77)	9.1 (5.08 to 13.11)
	*p*-value	0.979	0.878	0.049	0.001
	EMG vs. CNG	1.04 (−4.49 to 6.57)	−3.44 (−8.21 to 1.33)	−4.21 (−8.59 to 0.17)	−5.90 (−9.91 to −1.88)
	*p*-value	0.960	0.242	0.064	0.001
	PTG vs. EPG	0.89 (−4.64 to 6.42)	0.93 (−3.84 to 5.70)	2.97 (−1.41 to 7.35),	6.6 (2.58 to 10.61)
	*p*-value	0.974	0.956	0.293	0.001
	PTG vs. CNG	1.10 (−4.43 to 6.63)	−3.87 (−8.64 to 0.90)	−5.63 (−10.01 to −1.24)	−8.4 (−12.41 to −4.38)
	*p*-value	0.954	0.154	0.006	0.001
	EPG vs. CNG	0.21 (−5.32 to 5.74)	−4.80 (−9.57 to −0.02)	−8.6 (−12.98 to −4.21)	−15.0 (−19.0 to −10.9)
	*p*-value	1.000	0.048	0.001	0.001
Flexion rotation test (left side)	EMG vs. PTG	−0.09 (−5.49 to 5.31)	1.92 (−2.83 to 6.67)	1.90 (−2.35 to 6.15)	2.6 (−1.37 to 6.57)
	*p*-value	0.045	0.717	0.648	0.324
	EMG vs. EPG	0.9 (−4.50 to 6.30)	3.21 (−1.54 to 7.96)	5.7 (1.51 to 10.02)	9.54 (5.56 to 13.51)
	*p*-value	0.972	0.296	0.003	0.001
	EMG vs. CNG	0.8 (−4.6 to 6.20)	−1.5 (−6.25 to 3.25)	−4.89 (−9.14 to −0.63)	−5.92 (−9.89 to −1.94)
	*p*-value	0.980	0.842	0.017	0.001
	PTG vs. EPG	0.99 (−4.41 to 6.39)	1.29 (−3.46 to 6.04)	3.87 (−0.38 to 8.12)	6.94 (2.96 to 10.91)
	*p*-value	0.963	0.893	0.088	0.001
	PTG vs. CNG	0.89 (−4.51 to 6.29)	−3.42 (−8.17 to 1.33)	−6.79 (−11.04 to −2.53)	−8.52 (−12.49 to −4.54)
	*p*-value	0.973	0.243	0.001	0.001
	EPG vs. CNG	−0.10 (−5.50 to 5.30)	−4.71 (−9.46 to 0.04)	−10.66 (−14.91 to −6.4)	−15.4 (−19.43 to −11.48)
	*p*-value	0.955	0.053	0.001	0.001
Work ability	EMG vs. PTG	0.24 (0.83 to 1.31)	1.88 (0.55 to 3.20)	0.49 (−1.41 to 2.39)	2.28 (0.15 to 4.40)
	*p*-value	0.936	0.001	0.907	0.030
	EMG vs. EPG	0.20 (−0.87 to 1.27)	5.96 (4.63 to 7.28)	14.7 (12.79 to 16.60)	20.84 (18.71 to 22.96)
	*p*-value	0.962	0.001	0.001	0.116
	EMG vs. CNG	0.65 (−0.42 to 1.72)	−0.37 (−1.69 to 0.95)	−4.3 (−6.2 to −2.39)	−3.36 (−5.48 to −1.23)
	*p*-value	0.395	0.884	0.001	0.001
	PTG vs. EPG	−0.04 (−1.11 to 1.03)	4.08 (2.75 to 5.40)	14.21 (12.3 to 16.1)	18.56 (16.43 to 20.68)
	*p*-value	1.000	0.001	0.001	0.001
	PTG vs. CNG	0.41 (−0.66 to 1.48)	−2.25 (−3.57 to −0.92)	−4.79 (−6.69 to −2.88)	−5.64 (−7.76 to −3.51)
	*p*-value	0.751	0.001	0.001	0.001
	EPG vs. CNG	0.45 (−0.62 to 1.52)	−6.3 (−7.65 to −5.00)	−19.0 (−20.9 to −17.0)	−24.2 (−26.3 to −22.0)
	*p*-value	0.694	0.001	0.145	0.738

CgH, Cervicogenic Headache; EMG, Ergonomic modifications group; PTG, Physiotherapy group; EPG, Ergonomic modifications combined with physiotherapy group; CNG, Control group.

## 4 Discussion

Despite having recommendations in clinical practice guidelines (CPG) by Ontario protocol for traffic injury management (OPTIM) for ergonomic modification and physiotherapy for patients with CgH, no randomized controlled trial (RCT) has been conducted so far to find the individual and combined effects of these interventions on CgH patients (28). Based on the available literature, this is the first RCT conducted with the aim to investigate the combined and individual effects of workstation ergonomics, physiotherapy, and patient education in improving primary (CgH frequency) and secondary outcomes (CgH pain intensity, CgH disability, flexion rotation, and work ability) in office workers with cervicogenic headache. Recent studies report that the lower cervical spine (C5, C6, and C7) and upper thoracic spine (T1 and T2) are known to be one of the most mobile and functional regions in the human body. Therefore, stability is sacrificed at this junction and is more prone to joint dysfunctions. Patients with such joint dysfunctions may experience cervicogenic headache symptoms (29).

We hypothesized, that there is a difference in primary (CgH frequency) and secondary (CgH pain intensity, CgH disability, flexion rotation, and work ability) outcome measures between workstation ergonomics, physiotherapy, and patient education in office workers with cervicogenic headache and the results after 4 weeks of intervention and at 6 months follow up, within group analysis showed statistically significant changes in the primary (CgH frequency—89% improvement) and secondary (CgH pain intensity, CgH disability, and work ability) outcomes in all the four groups, but not in the flexion rotation for control group. In between group analysis, ergonomic modifications with the physiotherapy group (EPG) showed more significant improvement in decreasing the primary outcome CgH frequency (MCID = 11.9, 89%), when compared to EMG, PTG and the control group at 6 months’ follow-up. Similarly, CgH pain intensity and disability rate were also reduced in the EPG group (MCID = 4.6, 89%; 18.0, 86%) than the other treatment and control groups, which was supported by Shariat et al. (30) In addition the right and left side flexion rotation range (MCID = 15.0, 70%; 15.4, 69%) of the cervical region is increased in EPG group. So far, no studies have looked at the changes in work ability in office workers with CgH symptoms, but this study looked at the benefits of combined effects of ergonomic modifications with physiotherapy on work ability (MCID = 24.2, 80%) over isolated effects of ergonomic modifications and physiotherapy alone.

The ergonomics modifications included in the workstation, office environment and an ergonomics education to the study participants provided to the EMG and EPG showed significant improvements in CgH symptoms and work abilities of the participants. In our study, we made adjustments in the desk height to correct height for their shoulder girdle posture in relation to the desk. In addition, proper foot support was provided to relax the spine and lower extremity muscles. Also, a significant number of the study participants were sitting far away from the computer screen mainly due to the stable armrest in the chair, which causes them to lean forwards from their chair, resulting in a forward head posture. Therefore, they were advised to maintain a proper distance between the screen and the chair to reduce the strain on the neck muscles. Also, some workers kept their elbows on their desks which produces undue active tension on the shoulder muscles, hence, they were advised to sit back in the chair and to maintain an erect posture. Modifications were also done in the document holders, which should be more stable and large enough to accommodate the files. It is possible that these ergonomics workstation changes may have contributed to the significant improvements in CgH symptoms and work abilities, which is in agreement with Shariat et al. (30) and Lee et al. (16) A systematic review by Hoe et al., stated that ergonomics modifications are an instinctual way of decreasing the workload and thus preventing pain in work-related musculoskeletal disorders of the upper limb and neck among office workers (19). However, performing ergonomics modifications in the workstation is not an exact science, because each workstation and individual is slightly different with different challenges and these challenges become greater with different factors like limited sources, work pressure and low budget allotment.

According to our findings, physiotherapy in the form of manipulation of the cervical vertebra promotes afferent nerve fibre activity via joint receptors. It improves the overall action and properties of the neck muscles by activating the alpha motor neuron (31). It alters the sensory fibre activity by activating the joint receptors, thereby changing the *α*-motor neuron activity levels and subsequent muscle reaction. Because of the high mobility of the cervical spine, CSM can stimulate the receptors of deep neck muscles and sub-occipital muscles (32). Other theories for the pain-modulating effects of cervical manipulation included biomechanical, vertebral (temporal summation), and neural (central descending pain inhibitory pathway) mechanisms which were noted by Bialosky et al. (33) and Haavik-Taylor et al. (34).

More significant improvements were reported in all the outcome measures in the combined treatment group (EPG) than in the other groups. It may be due to the combined effects of ergonomic modification and physiotherapy which was in agreement with Tsang SMH et al. (15) but Pillastrini et al. (35) reported that such an intensive intervention may not be financially viable in the long term. The little changes noted in the control group (patient education) on pain frequency and other outcome variables explained the psychological and analgesic effect of patient education on cervicogenic headache. It facilitates the anti-nociceptive response by activating the opioid and oxytocin interaction through the release of neurotransmitters by stimulating the local nerves. The findings of this trial would assist physiotherapists in making decisions to select the best approach for CgH patients.

### 4.1 Clinical implications

The study’s findings carry significant implications for researchers, clinicians and patients. For researchers, it offers insights into the early and advanced stages of cervicogenic headache. Clinicians can use this knowledge to enhance screening protocols and implement timely interventions, potentially improving patient outcomes. Patients stand to benefit from early detection and tailored preventive measures, mitigating long-term complications of cervicogenic headache. Ultimately, this study underscores the importance of ergonomics and physiotherapy in office workers with cervicogenic headache symptoms.

### 4.2 Limitations

The study had some limitations during its execution, which should be considered for future studies. First, the study included both genders, but the reports collected were not calculated independently during the statistical analysis, these differences may influence the research reports. Second, it is impossible to ensure that the subjects completed the log book daily rather than after a week or 4 weeks. Third, this study lacks continuous monitoring, as some participants may change the workstation modifications which may affect the study findings. Fourth, the other confounding parameters such as duration of sitting, frequency of movement, and physical activity were not measured. Finally, the treatment preference of physiotherapists and patients was not asked which could have affected the results. Future studies are recommended to find the biomechanical changes underlying these clinical and functional changes of these techniques in treating patients with CgH symptoms.

## 5 Conclusion

The reports of this current randomized clinical study observed that the workstation ergonomics and physiotherapy group experienced significantly more improvements in cervicogenic headache (frequency, intensity and disability), functional range of motion and work ability than ergonomic modification, physiotherapy and patient education alone in office workers with cervicogenic headache symptoms.

## Data Availability

The raw data supporting the conclusions of this article will be made available by the authors, without undue reservation.
